# Optic neuritis, the most common initial presenting manifestation of multiple sclerosis in northern Iran

**Published:** 2015

**Authors:** Seyed Mohammad Masoud Hojjati, Amin Zarghami, Seyed Ali Hojjati, Mobina Baes

**Affiliations:** 1 Department of Neurology, Babol University of Medical Sciences, Babol, Iran.; 2 Student Research Committee, Babol University of Medical Sciences, Babol, Iran.; 3 Shahid Beheshti University of Medical Sciences, Tehran, Iran.; 4 Babol University of Medical Sciences, Babol, Iran.

**Keywords:** Multiple sclerosis, Optic neuritis, Iran.

## Abstract

**Background:**

Multiple sclerosis (MS) is an inflammatory and demyelinating disease of central nervous system (CNS). The aim of the present study was to determine the type and the frequency of initial presenting symptoms in patients with MS and their relation with demographic characteristics in Babol, northern Iran.

**Methods:**

All patients of this study were recruited over a ten year period from 2002 to 2012 from single neurologic clinic. Diagnosis of MS was confirmed according to the McDonald criteria, demographic and clinical features Then, all the clinical findings and demographic variables including: age, sex, marital status, age at onset, education, place of residence, disease duration, initiation pattern of disease have been collected. Expanded Disability Status Scale (EDSS) was used for the evaluation of disability at the onset of disease. Data analysis was performed by chi-square test.

**Results:**

A total of 263 consecutive MS patients with the age range of 17 to 61 yr were examined. Optic neuritis was the most prevalent initial presenting symptom in 123 (46.8%) patients followed by sensory disturbances as the second common presenting symptom of MS. Significant difference was found between patients with or without optic neuritis and the onset age of the disease and EDSS (p<0.001). The mean EDSS score at the time of initial presentation was 1.67±0.77.

**Conclusion:**

The findings of this study indicated that optic neuritis is the most prevalent initial presentation of MS in the geographic region of northern Iran. In patients less than 30 years, development of visual disturbances justifies neurologic examination.

Multiple sclerosis(MS) is a chronic inflammatory demyielinating disease that attacks myelinated axons in the central nervous system, damaging the myelin and the axon in variable degrees and producing significant progressive physical disability in patients (1). The hallmark of MS is symptomatic episodes occurs in months or years apart and involves various anatomic locations. The etiology of MS is unknown; but it seems the combination of genetic susceptibility and environmental factors (eg. viral infection, low vitamin D levels, etc.). Has a contribution in the development of MS Geographic variation in the incidence of MS supports the probability that environmental factors are involved in the etiology (2-3). More than 2.1 million people are affected by MS worldwide (4). MS is more common in women and the estimated incidence for the female to male ratio reached to 2.3 in 2000 (5). Although MS can occur in any age groups, it usually diagnosed in persons aged 15-45 years (6).

The disease seen in all parts of the world and in all races; but rates vary widely. Based on the Kurtzke classification, Iran is located in a low-risk zone for MS (7). However, recent studies on the prevalence of MS came up with different results and indicated a higher prevalence rate in our country (8-9). In previous studies in various countries, different frequencies reported for initial presenting symptoms of MS. Concerning the sites of involvement, there have been several reports that in eastern MS patients more commonly represent clinical evidence of more involvement of the optic nerves and spinal cord than Caucasians (10). Optic neuritis is one the common presenting symptoms of MS which developed due to the involvement of optic nerve in the process of pathogenicity of the MS. This complication causes the deficit in visual acuity mainly in one of the eye. Depending on which part of the nerve is affected and the area of demyelination, manifestations of MS can vary apparently from benign condition to incapacitating clinical symptoms (11). 

Previous studies in recent decades indicated higher frequency of optic neuritis in Iranian patients with MS as compared with other countries (12, 13). Nonetheless, the data regarding the associated factors as well as the types and the frequency of the initial presenting manifestation of MS in Iran is scarce. Thus, the aim of this study was to assess the type and frequency of initial presenting symptoms of MS with regard to age at disease onset, sex and demographic features in geographic region of Babol, Northern Iran.

## Methods

In this cross-sectional study we reviewed all the 263 consecutive MS patients who had been admitted to a Multiple Sclerosis Center from 2002 to 2012 in Babol, northern Iran. Although the majority of the registered patients are residents of Babol, a city in North of Iran but a number of patients were referred from other cities of Mazandaran. The McDonald criteria applied in the present study and data confirmed by a neurologist in order to diagnose MS in all the consecutive patients (14). The protocol of the study was approved by the ethical board of the research council of Babol University of medical sciences. Data were collected for demographic and clinical features of MS such as the duration of disease, age at onset of first symptom, family history, and associated factors of optic neuritis. The authors were committed to the ethical consideration during the stages of the study and follow the Declaration of Helsinki. 

After receiving the informed consent, all patients underwent a standard neurologic examination. The main manifestations assessed by the investigators based on impairment of sensory disturbance, impairment of cranial nerves, impairment of motor pathways, cerebellar symptoms (tremor, dysarthria etc.), impairment of visual pathways, brain stem function, impairment of bladder or sexual dysfunction. The diagnosis of optic neuritis was made by the recommendations for clinical practice of the International Federation of Clinical Neurophysiology (15). All patients underwent a complete ophthalmic examination. Neurologists diagnosed patients’ optic neuritis according to the standard methods. We used Kurtzke Expanded Disability Status Scale (EDSS) for the evaluation of disability in patients with MS (16). Data was analyzed by SPSS Version 18 (SPSS Inc., Chicago, Ill., USA) and chi-square test for comparison of the proportions. A p-value less than 0.05 was considered significant.

## Results

**Figure 1:**
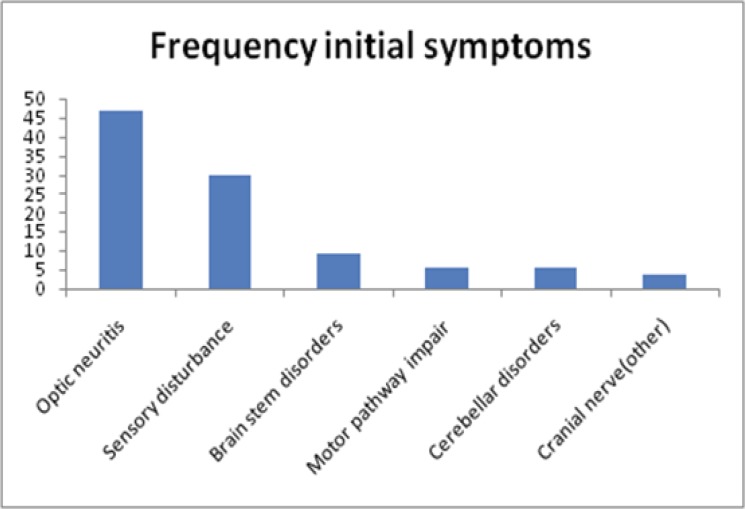
Frequency of initial symptoms in 263 MS patients

A total of 263 consecutive patients with MS were examined (28.5% men; 71.5% women). The mean age of participants was 34.28±9.47 (age range, 17 to 61 yr). The female to male ratio was 2.5. The course was relapsing-remitting (RR) in 91% of patients and primary progressive (PP) in 9%. Optic neuritis with the prevalence of 46.8% was the most prevalent initial presenting symptoms in our patients with MS. Figure 1 shows the prevalence of different initial presenting symptoms in our patients with MS. 

Monosymptomatic initiation pattern was seen in (161.263) 61.2% of the cases. The mean EDSS at the onset of disease was 1.67±0.77. The distribution of demographic information and features of the disease among MS patients presented with optic neuritis and without it are presented in Table 1. Significant difference was found between patients with or without optic neuritis and onset age of the disease (p<0.001); however in other variables such as gender, marital status, education, place of live and initiation pattern did not indicated any significant differences.

**Table 1:** Demographic and clinical profile of 263 patients with Multiple Sclerosis with or without optic neuritis

**Variables**	**Optic neuritis**		**Odds Ratio**	**95%** **Confidence interval**		**P value**
	**With** **(n=160)**	**Without** **(n=103)**		**Lower**	**Upper**	
**Gender** Male Female	39 (24.4%) 121 (75.6%)	36 (35%) 67 (65%)	1.66	0.96	2.86	0.06
**Marital status** * Single married	47 (29.7%) 111 (70.3%)	22 (21.4%) 81 (78.6%)	0.64	0.35	1.14	0.13
**Place of live** Urban Rural	93 (58.1%) 67 (41.9%)	52 (50.5%) 51 (49.5%)	0.73	0.44	1.20	0.22
**Education** Lower diploma Higher diploma	98 (61.3% 62 (38.8%)	66 (64.1%) 37 (35.9%)	1.12	0.67	1.88	0.66
**Age at onset** ≤30 >30	130 (81.3%) 30 (18.8%)	63 (61.2%) 40 (38.8%)	0.36	0.20	0.63	<0.001
**Initiation pattern** Monosymptomatic Polysymptomatic	101 (63.1%) 59 (36.9%)	60 (58.3%) 43 (41.7%)	0.815	0.491	1.35	0.42

* The data of two cases was missing.

## Discussion

According to our findings, optic neuritis was observed in 46.8% of the patients with MS as the most prevalent initial presenting symptom in our study population. But in contrast to similar studies in Iran, our data placed in the row of high frequencies.

 Saadatnia et al. reported that the most common symptom at the first episode was sensory disturbance (51.1%), followed by optic neuritis (47%) (13). 

In our study 61.2% of the patients with optic neuritis - as a presenting symptom- had monosymptomatic initiation pattern of the disease. There were limited studies regarding to optic involvement in related to other initiation patterns of the disease. Several reports have demonstrated that monosymptomatic onset occurs in adults than in childhood (17, 18). Ruggieri et al revealed that brain stem syndromes or cerebellar ataxia were the most common onset symptom in monosymptomatic children (19). While in a population-based study in Isfahan-Iran the most common onset symptom, optic neuritis (31%) was the most frequent initial presentation in early onset MS and polysymptomatic onset was more common in early-onset patients (35.6%) compared to adult-onset patients (12%) (17). In our study optic neuritis involvement in patients with the age of lower than 30 at onset of the disease was significantly more than the patients more than 30 years old. Ashtari et al. investigated the characteristics of early-onset (EOMS) Multiple Sclerosis in comparison to adult-onset (AOMS) in Isfahan, Iran. They revealed that the most common presenting symptom was optic neuritis in the EOMS patients and paresthesia in AOMS despite of no significant difference in terms of major clinical manifestations (20). 

However, it is assumed that in different populations with different social and individual characteristics, the distribution of MS might differ which leads to various reasons like genetic, environment and geographical location (21, 22). In a study of comparing the role of ethnicity on the characteristics of MS among Persians and Iranian- Arab population, the authors indicated that optic nerve(40%) involvement was the most frequent features of initial symptoms which followed by sensory problems in Persians. On the other hand, motor deficit and cerebellar symptoms are more prevalent among Iranian- Arabs than Persians(19.1% and 18.2% versus 8.5% and 9.8% respectively( (23). While reports from several Arab countries revealed that motor dysfunction was the most prevalent feature of initial presentation (24, 25). For example, Sidhom et al. in Tunisia reported motor symptoms (28%) followed by optic neuritis (20%) as the most frequent isolated onset symptoms (26). According to the close neighboring of these countries, the differences are the matter of controversy. These contrasts attributed to the socioeconomical differences rather than genetic and ethnicity(27). Reports from Latin America also revealed that motor dysfunction followed by optic neuritis and sensory dysfunction were the most prevalent initiating symptoms (28). In a study in Bengal, India based on Poser's criteria the Visual impairment (53.33%), weakness of limbs (31.11%) and sensory paresthesia (20%) were the common presenting symptoms (29). Studies from China and eastern Asia were revealed controversial frequencies of sensory disturbance, visual loss and optic neuritis (30, 31). The results of this study should be considered with limitations. Since the study population has been collected over a period of 10 years, therefore the spectrum of clinical features and the nature of MS are expected to be changed compared with new cases. Nontheless, this study has are strength with regard to diagnostic method using a single diagnostic criteria, as well as patient characteristics which have been recruited from single neurologic clinic. The findings of this study is clinically important. Because, vitamin D deficiency has been suspected as a possible environmental risk factor of MS and vitamin D deficiency is highly frequent in the geographic locale of the study subjects particularly the young women (32). Several studies have shown an association between vitamin D deficiency and several skeletal and nonskeletal conditions (33-35). This issue justifies a study regarding to MS and vitamin D deficiency. 

In conclusion, this study indicated that optic neuritis is the most prevalent initial presenting symptoms of MS in the geographic region of Babol. Based on the results of the present study, development of ophthalmic symptoms in young patients justify neurological evaluation particularly for MS.

## Notes

**Funding: **This study was funded by the Chancellor for Research and Technology (Grant no: 2834).
